# An Account of the Philadelphia College of Pharmacy

**Published:** 1842-04-09

**Authors:** Joseph C. Turnpenny


### *An Account of the Philadelphia College of Pharmacy.*

By Joseph C. Turnpenny.

This valuable institution, the first of its kind in this country, was established in the year 1821, by a number of respectable Druggists and Apothecaries for the promotion of the sciences of Chemistry, Materia Medica, and Pharmacy, and the year following was incorporated under the above title.

The necessity for druggists and apothecaries co-operating together at that time was obvious from the fact that, at a meeting of the Board of Trustees of the University of Pennsylvania, a resolution was passed on the 6th of February, 1821, for the purpose of conferring the degree of Master of Pharmacy upon any one who had complied with the following requisitions, viz.: To serve an apprenticeship of three years to a reputable druggist or apothecary; to attend two courses of lectures, in the University, on Chemistry, Materia Medica, and Pharmacy; and also to produce from his employer a certificate of his good moral character.

At a meeting of druggists and apothecaries assembled about that time, for the purpose of taking into consideration the above resolution, we find that they rejected this offer of the University, and formed themselves into an association for the purpose of having some control over the interests of the trade in this city. The following is part of a report introduced at one of the first meetings of the College:

"A very general impression appears to have prevailed amongst the druggists and apothecaries of this city for some time past, that, from a concurrence of various circumstances, a departure from the correct customs and established principles of the drug and apothecary business has in some instances taken place; [and] as a consequent effect, the deterioration of many drugs and medicines in constant use, and of great importance in practice, and [that] medicines of inferior or sophisticated qualities: are too often introduced into the shops. The want of proper pharmacological information on the part of some druggists and apothecaries who vend, and of physicians who buy, has mainly tended to the production of these irregularities."

To the founders of this institution too much praise cannot be awarded for their untiring zeal in originating a plan which has been of such incalculable advantage in elevating the standard of pharmaceutical knowledge.

With the foundation of the College commenced a new era in Pharmacy on this side of the Atlantic. Our stores were formerly supplied chiefly from Europe, and every new principle or remedy sought for from thence, while we were standing comparatively idle, and ignorantly waiting the results of their experiments.

In the year 1825, a Journal was commenced under the immediate auspices of the College, which became in the year 1829 a quarterly Journal, under the editorship of the late Dr. Benjamin Ellis; since which time the Journal has disseminated much valuable information.

Two courses of lectures are delivered in this institution in the winter months; one on Chemistry, the other on Materia Medica and Pharmacy. A student, before he is eligible for graduation, is required to attend two courses of each of these lectures; to have served an apprenticeship of four years, and to produce from his employer a certificate of his good moral character, and also a dissertation on some subject connected with Chemistry, Pharmacy, or Natural History. After an examination before the professors, and a committee of three members, if found competent, he is proposed to the Board of Trustees, whose duty it is to declare him a graduate in the Philadelphia College of Pharmacy.

Every facility is afforded to the student in this institution. Attached to it is a library of upwards of five hundred volumes, a cabinet of specimens, and various chemical apparatus. In addition to the ordinary sessions, conversational meetings are held semi-monthly during the winter.

The College owes much of its reputation to the following gentlemen, who have occupied its professorial chairs since its commencement: Drs. S. Jackson, Geo. B. Wood, B. Ellis, F. Bache, R. E. Griffith, J. Carson, and Wm. R. Fisher. The two last are the present incumbents, and it affords us much pleasure to state that the latter gentleman was a graduate in this institution.

The number of graduates since its commencement has been eighty-five. Of these, many are distributed over the United States, repaying their alma mater for the care extended in their professional education, by reflecting upon her the honor of their exertions.

